# Every Man to His Trade

**Published:** 1894-10

**Authors:** 


### Every Man to His Trade.

I know a woman who has five doctors whom she consults at regular intervals. Number one, who is of most importance, looks after her general system, and is consulted when the trouble cannot be definitely located. Number two is the eye, ear, and throat specialist. Number three takes care of her skin and complexion. Number four has tackled her hæmorrhoids, but has not cured them; he is the "rectologist." Number five, ah, number five! he is in the swim just now; he palpates the appendix, the ovaries and their accessories, and is ready for a laparotomy should a pain strike his territory. I know she has also been in the hands of the dentist and chiropodist.—*Louisville Medical Monthly.*

